# Enhancing dementia care practice with Be EPIC‐VR: An implementation science approach

**DOI:** 10.1002/alz70858_101298

**Published:** 2025-12-25

**Authors:** Marie Y. Savundranayagam, Annette Schumann, Grace Norris, Jennifer L. Campos, Joseph B Orange

**Affiliations:** ^1^ Western University, London, ON, Canada; ^2^ KITE ‐ Toronto Rehabilitation Institute, University Health Network, Toronto, ON, Canada

## Abstract

Be EPIC‐VR is a virtual reality program that trains frontline dementia care providers improve their person‐centered communication skills. This study used the Consolidated Framework for Implementation Research to examine how factors in the outer setting, inner setting, and innovation domains supported the successful implementation of Be EPIC‐VR into home care and long‐term care settings.

Eight managers from four care settings in Ontario were selected for their decision‐making roles in facilitating frontline staff engagement with Be EPIC‐VR. Semi‐structured interviews were conducted at three intervals (pre‐intervention, two weeks post‐intervention, and five months post‐intervention) to identify factors impacting Be EPIC‐VR's implementation. The resulting data were analyzed using framework analysis.

Three main themes emerged across all data collection intervals: 1) the need for dementia‐specific training, 2) intervention alignment with organizational culture, and 3) openness to VR as a training tool. Theme 1, the need for dementia‐specific training, stemmed from external pressures (i.e., outer setting) to maintain a highly skilled workforce and tension for change within organizations (i.e., inner setting) driven by gaps in training on person‐centered communication and addressing responsive behaviors. Post‐implementation, managers highlighted Be EPIC‐VR's relative advantage (i.e., innovation domain) in meeting gaps identified pre‐implementation. Theme 2, intervention alignment with organizational culture, reflected inner setting norms that prioritize staff education, resident‐focused care, and staff competence in serving diverse populations. These factors remained relevant through all time points. Finally, for theme 3 on openness to VR as a training tool, managers initially pointed to inner setting factors, including resource requirements, workflow compatibility of the innovation, and accommodation for less tech‐savvy staff. Post‐implementation, managers highlighted Be EPIC‐VR's adaptability to organizational processes by using small‐group sessions and flexible scheduling, its compatibility through remote delivery that fit current workflows, and its relative advantage of providing facilitator support.

Successful implementation depended on aligning Be EPIC‐VR with organizational goals, staff workflows, and staff competencies. Building adaptability into the implementation process and offering direct support were critical in fostering openness to VR‐based training. By addressing organizational readiness and tailoring resources, Be EPIC‐VR holds promise for improving dementia care through enhanced communication skills.

